# A repository of assays to quantify 10,000 human proteins by SWATH-MS

**DOI:** 10.1038/sdata.2014.31

**Published:** 2014-09-16

**Authors:** George Rosenberger, Ching Chiek Koh, Tiannan Guo, Hannes L. Röst, Petri Kouvonen, Ben C. Collins, Moritz Heusel, Yansheng Liu, Etienne Caron, Anton Vichalkovski, Marco Faini, Olga T. Schubert, Pouya Faridi, H. Alexander Ebhardt, Mariette Matondo, Henry Lam, Samuel L. Bader, David S. Campbell, Eric W. Deutsch, Robert L. Moritz, Stephen Tate, Ruedi Aebersold

**Affiliations:** 1 Department of Biology, Institute of Molecular Systems Biology, ETH Zurich, CH-8093 Zurich, Switzerland; 2 PhD Program in Systems Biology, University of Zurich and ETH Zurich, CH-8093 Zurich, Switzerland; 3 Ruprecht Karls University of Heidelberg, DE-69117 Heidelberg, Germany; 4 PhD Program in Molecular and Translational Biomedicine, Competence Centre for Systems Physiology and Metabolic Diseases (CC-SPMD), University of Zurich and ETH Zurich, CH-8093 Zurich, Switzerland; 5 Department of Phytopharmaceuticals (Traditional Pharmacy), School of Pharmacy and Pharmaceutical Sciences Research Center, Shiraz University of Medical Sciences, 71345-1583 Shiraz, Iran; 6 Division of Biomedical Engineering and Department of Chemical and Biomolecular Engineering, The Hong Kong University of Science and Technology, Clear Water Bay, Hong Kong, China; 7 Institute for Systems Biology, Seattle, Washington 98109-5234, USA; 8 AB SCIEX, Concord, Ontario L4K 4V8, Canada; 9 Faculty of Science, University of Zurich, CH-8057 Zurich, Switzerland

## Abstract

Mass spectrometry is the method of choice for deep and reliable exploration of the (human) proteome. Targeted mass spectrometry reliably detects and quantifies pre-determined sets of proteins in a complex biological matrix and is used in studies that rely on the quantitatively accurate and reproducible measurement of proteins across multiple samples. It requires the one-time, *a priori* generation of a specific measurement assay for each targeted protein. SWATH-MS is a mass spectrometric method that combines data-independent acquisition (DIA) and targeted data analysis and vastly extends the throughput of proteins that can be targeted in a sample compared to selected reaction monitoring (SRM). Here we present a compendium of highly specific assays covering more than 10,000 human proteins and enabling their targeted analysis in SWATH-MS datasets acquired from research or clinical specimens. This resource supports the confident detection and quantification of 50.9% of all human proteins annotated by UniProtKB/Swiss-Prot and is therefore expected to find wide application in basic and clinical research. Data are available via ProteomeXchange (PXD000953-954) and SWATHAtlas (SAL00016-35).

## Background & Summary

Much of science depends on reproducible and quantitatively accurate measurements. In the molecular life sciences, technological advances have moved the large-scale measurement of the molecules that constitute living cells to the forefront. For example, next generation sequencing (NGS) technology has made the routine quantitative analysis of complete genomes and transcriptomes a reality in many laboratories. In contrast, the analysis of proteins, the predominant class of functional effector molecules of the cell, has remained challenging and not generally accessible.

In most laboratories, proteins in complex samples are detected and quantified via immunoassays where specific reagents, frequently antibodies, are used to generate a signal that indicates the presence and quantity of a specific protein in a sample. Large-scale programs, exemplified by the Human Protein Atlas project^1^ and commercial efforts have attempted to generate specific affinity reagents for each human protein and to make them widely accessible. Undoubtedly, the availability of these reagents has the potential to significantly impact life science research. At present, however, only a subset of the proteome is routinely measurable by affinity reagents, with the consequence that much of the literature knowledge about proteins is focused on a relatively small subset of the proteome, the fraction for which affinity reagents are readily available^2^. Furthermore, at least some of these reagents are of unknown and dubious quality^3^, limiting the utility of the results obtained. Therefore, life science research would greatly benefit from the general availability of validated, high quality assays for the human proteome.

Mass spectrometry (MS) has become the method of choice for the deep and reliable exploration of the (human) proteome. In particular, liquid chromatography-coupled tandem mass spectrometry (LC-MS/MS) operated in data-dependent acquisition mode (DDA), has achieved remarkable progress in the identification of proteins in complex samples. Proteome-wide identification and quantification have been achieved for human cell lines^4–6,4–6,4–6^ and efforts are being made to characterize at least one protein product of all 20,300 protein-coding genes. An example of such an effort is the HUPO Chromosome-centric Human Proteome Project^7^, which could detect at least one single peptide for~14,000 proteins to date^8^. Recently, two independent studies from Kim *et al.*^9^ and Wilhelm *et al.*^10^ reported the cumulative analysis of more than 2,000 and 16,800 LC-MS/MS measurements, respectively, that yielded a map of identified peptides corresponding to 17,294 and 18,097 human protein-coding genes, respectively. However, the high degree of proteome coverage achieved in these studies depends on protein or peptide fractionation techniques like strong anion exchange (SAX) or off-gel electrophoresis (OGE) prior to MS analysis, to distribute the sample complexity among several instrument injections and the integration of the results of a high number of LC-MS/MS measurements. The high technical complexity and cost of generating and analyzing deep proteomic datasets and well understood technical tradeoffs^11^ have so far prohibited the distribution of this powerful technology to a large number of laboratories^12^ and limited the reproducibility of datasets generated within and across laboratories^13–15,13–15,13–15^ thus limiting the breadth of its impact.

We and others have proposed that targeted mass spectrometry has the potential to democratize mass spectrometry-based proteomics, i.e., to make most or all proteins reliably detectable and quantifiable in a large number of laboratories^16^. Under the umbrella of the HUPO Human Proteome Project^17^, we launched a program to make the targeting technology and associated measurement assays generally accessible. In targeted proteomics, exemplified by the prototypical quantitative MS technique selected reaction monitoring (SRM), also referred to as multiple reaction monitoring (MRM), predetermined sets of proteins are accurately quantified by means of specific mass spectrometric assays that have to be generated *a priori* once for each targeted protein. In support of SRM-based protein quantification, extensive, in some cases proteome-wide, assay libraries and empirical measurements of the same assays across multiple samples to judge performance of these assays have been created^18–21,18–21,18–21,18–21^ and made freely accessible (http://www.srmatlas.org, http://www.peptideatlas.org/passel/). While SRM and the recent implementations of the related method parallel reaction monitoring (PRM) on high performance mass spectrometers^22^ remain the best performing quantitative MS methods, they are limited by the relatively low number of proteins (50–100) that can be quantified in a single injection and the fact that the targeted proteins need to be specified for each sample prior to data acquisition.

Recently, we introduced SWATH-MS, a new mass spectrometric technique that combines data-independent acquisition (DIA) with targeted data extraction on a high resolution mass spectrometer^23^. In DIA mode, the instrument deterministically fragments all precursor ions within a predefined mass-to-charge (*m/z*) range and acquires convoluted product ion spectra, containing the fragment ions of all concurrently fragmented precursors. By rapidly and recursively scanning through consecutive, adjacent precursor ion windows, termed swathes, the full precursor ion *m/z* range of trypsinized peptides is covered and consequently, fragment ion spectra of all precursors within a user defined retention time (RT) versus *m/z* window are recorded over time. This results in a data set that is continuous in both fragment ion intensity and retention time dimensions and essentially represents a digital recording of the protein sample analyzed. Within these data, specific peptides can be identified and quantified by applying a targeted data extraction strategy that results in signals analogous to those obtained by SRM, where sets of fragment ion signals uniquely associated with the targeted peptide are recorded over chromatographic time and the concluding peak groups are used as evidence for the conclusive identification and quantification of the targeted peptide in a sample. The data analysis depends on *a priori* assays, derived from fragment ion spectra of the targeted peptides that are best generated in the same high resolution instrument used for SWATH-MS acquisition. In contrast to SRM where the targeted peptides need to be determined prior to data acquisition, SWATH-MS datasets are recorded independently and can then be perpetually re-mined using the targeted analysis strategy. Using freely or commercially available software (OpenSWATH^24^, Skyline^25^, PeakView (AB SCIEX, Concord, Canada) or Spectronaut (Biognosys AG, Schlieren, Switzerland)) and a proteome-specific assay library, SWATH-MS can be used to carry out protein quantification at performance metrics that are comparable to SRM but at a much higher throughput^23,24^.

To date, most studies using SWATH-MS have relied on the generation of sample-specific assay libraries, acquired in fractionated or enriched samples, injected prior to SWATH-MS acquisition on the same instrument operated in DDA mode^23,24,26–29,26–29,26–29,26–29^. Here we present a generic large-scale human assay library to support protein quantification by SWATH-MS. It is optimized for targeted data analysis of SWATH-MS data sets acquired on AB SCIEX TripleTOF 5600+ Systems. It consists of 1,164,312 transitions identifying 139,449 proteotypic peptides and 10,316 proteins. It was generated by combining the results from 331 measurements of fractions from different cell lines, tissue and affinity enriched protein samples. The assays consist of precursor and fragment ion *m/z*, normalized RT and relative ion intensities, making this resource readily applicable for data analysis using state-of-the-art analysis software. We further demonstrate that the results and biological conclusions obtained with the combined assay library are comparable to those obtained with sample-specific assay libraries and applicable across laboratories. We expect that this resource will contribute significantly to the simplified and reproducible analysis of human proteome samples across studies and laboratories.

## Methods

### Sample overview

**Figure 1:**
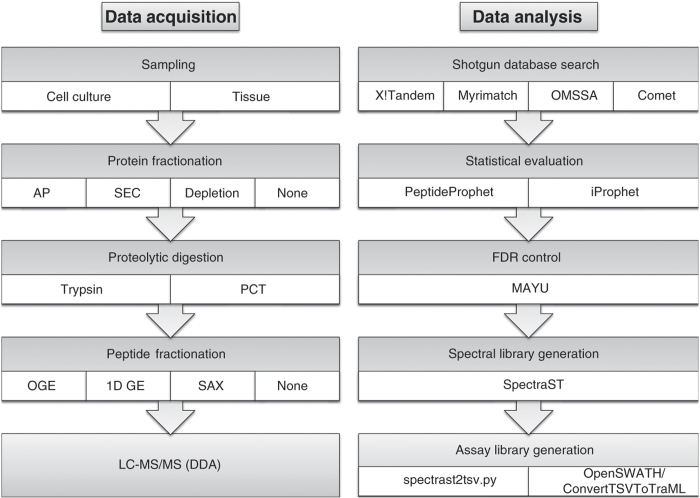
Data acquisition and data analysis workflows employed for the generation of assay libraries. (**a**) Data acquisition: Sampling of different cell lines and tissue types was followed by (optional) protein fractionation, proteolytic digestion (using trypsin or lys-c/trypsin using PCT), (optional) peptide fractionation and LC-MS/MS analysis in discovery proteomics mode. (**b**) Data analysis: Sequence database search was conducted using four different search engines and the results were statistically evaluated and combined using the Trans-Proteomic Pipeline. False discovery rate (FDR) control was conducted using MAYU. The identified peptides were used to generate a consensus, RT normalized spectral library using SpectraST. Assays were selected using spectrast2tsv.py and the OpenSWATH tool ConvertTSVToTraML.

To achieve broad representation of the human proteome we analyzed protein samples from a range of human cell and tissue types. The specific sample types analyzed are summarized in Table 1 (for complete annotation see Supplementary Table 1) and include human cell lines, tissues such as kidney, gut, monocytes, neutrophils and human blood. To increase the contents of the assay library of proteins of low abundance, we also added spectra obtained from affinity purified protein complexes. Figure 1 illustrates the experimental workflow.

### Cell culture, tissue sampling and protein-level separation

#### Cell culture

HEK293 cell samples were essentially generated as described before^30^.

HeLa and U2OS cells were obtained from ATCC and grown in DMEM with GlutaMAX-1 (Invitrogen, Carlsbad, CA) supplemented with 100 U/ml penicillin, 100 μg/ml streptomycin and 10% fetal bovine serum at 37 °C, 5% CO_2_, in a humidified incubator.

NCI60 and LNCaP cells were obtained as frozen, non-viable cell pellets from the Developmental Therapeutics Program (DTP), National Cancer Institute (NCI NIH).

CAL51 cells were grown in RPMI 1640 media depleted of arginine and lysine (Invitrogen) and supplemented with 10% Fetal Bovine Serum (Invitrogen, 26400-044) (FBS). The media was supplemented with 100 U/ml penicillin, 100 μg/ml streptomycin, 2 mM L-glutamine (Gibco). THP1 cell line samples were generated as described before^31^.

#### Patient specimen

Kidney tissue samples (*n*=18) were collected at the time of surgery and were provided by Dr Silke Gillessen, Dr Markus Joerger and Dr Wolfram Jochum (Kantonsspital St Gallen, Switzerland).

Gut tissue samples (*n*=18) were provided by Dr Marko Kalliomaki (Turku University Hospital, Finland). The samples were collected during the diagnostic colonoscopy from nine patients.

Lung tissues samples (*n*=12) were provided by Dr Wim Timens and colleagues at the University Medical Center Groningen, Netherlands.

Muscle tissue samples (*n*=12) were provided by Dr Carsten Jacobi (Novartis Pharma AG, Switzerland).

Blood plasma samples were obtained from 32 female healthy donors and mixed together before further processing. Plasma was depleted of the 14 most abundant plasma proteins with the multiple affinity removal system (MARS Hu-14 spin cartridge; Agilent Technologies) according to the manufacturer’s protocol. Depleted samples were exchanged with a 3,000 Da molecular weight cutoff filter (Pall Corporation) and denatured in 6 M urea and 0.1 M ammonium bicarbonate before digestion with trypsin and LC-MS analysis.

Monocytes & neutrophils samples were isolated from patients with active tuberculosis and were provided from Prof Dr Stefan Kaufmann (Max Planck Institute for Infection Biology, Berlin, Germany).

Purified platelets from a healthy donor were provided by Prof. Ohad Medalia (University of Zurich, Switzerland). The purification and protein digestion were performed essentially as described before^32^.

All clinical specimens were obtained under IRB approval and accepted protocols. Written informed consent was obtained from all patients from whom biopsy samples were taken.

#### Affinity purification

Previously published datasets from affinity purification samples of the 14-3-3 beta network were included^27^. In addition, pull-downs of human kinase baits according to the same protocol were generated and pooled for the purpose of spectral library generation.

#### Size-exclusion chromatography (SEC)

Cycling HEK 293 wt cells were lysed essentially as described before^27^, except that the lysis buffer was not supplemented with avidin. Lysates were cleared by 15 min of ultracentrifugation (100,000×*g*, 4 °C, Beckman Coulter Optima TLX ultracentrifuge) and lysis buffer was exchanged to SEC buffer (50 mM HEPES pH 7.5, 150 mM NaCl) over 30 kDa molecular weight cutoff membrane (Amicon Ultra-15, Millipore, MA, USA), at a ratio of 1:50 in three dilution and re-concentration steps of 1:2, 1:5 and 1:5. Proteins were concentrated to 25–30 mg/ml as judged by OD280 and were then cleared from precipitates by 5 min of centrifugation at 16.9 krcf at 4 °C (Eppendorf 5,418R) before protein level fractionation. SEC was performed on an Agilent 1,100 milliliter flow HPLC system (Agilent, CA, USA) utilizing a Yarra-SEC-4000 column (pore size 500 Å, dimensions 300×7.8 mm, Phenomenex, CA, USA) in 50 mM HEPES pH 7.5, 150 mM NaCl with temperature controlled at 4 °C and at a flow rate of 500 ul/min. 1 μg of concentrated lysate was injected for fractionation into 80 fractions collected from 10–25 min post-injection. Two consecutive runs were pooled to yield the final set of fractions for digestion and analysis via LC-MS/MS.

### Peptide sample preparation for MS

To maximize the proteome coverage of the individual specimen, the samples were fractionated using different physicochemical methods like off-gel electrophoresis or ion exchange chromatography. In this study, we included SEC and OGE fractionated samples from a HEK293 cell line, SAX fractionated samples from plasma and LNCaP cell lines and OGE fractionated samples from THP1 and NCI60 cell lines.

#### Proteolytic digestion

The protein samples were reduced with 5 mM TCEP, and alkylated with 10 mM iodoacetamide before overnight trypsinization. Some samples were trypsinized using the Pressure Cycling Technology (PCT) protocol as described below (indicated in Table 1). Protein from SEC fractions was denatured by incubation at 69 °C for 10 min, reduced, alkylated and digested in the presence of 1% (v/v) Sodium-deoxycholate overnight. Trypsin was inactivated by lowering the pH to 2 and the peptides were immobilized onto C18 columns. After multiple washes, the peptides were eluted (50% acetonitrile/0.1% formic acid) and solvents were evaporated in a SpeedVac centrifuge. After re-suspension, the samples were briefly sonicated before MS analysis.

#### PCT-assisted lysis and digestion

Pressure cycling technology (PCT)^33^ applies cycles of hydrostatic pressure between ambient and ultra-high levels to induce cell lysis and to enable precise thermodynamic control of biomolecular interactions. All PCT-processed samples were handled using Barocycler® NEP2320 (PressureBioSciences, Inc, South Easton, MA). In brief, tissue or cell line samples were lysed in buffer containing 8 M urea, 100 mM ammonium bicarbonate supplemented with Complete protease and phosphatase inhibitor cocktail under Barocycler program (tissue samples: 60 cycles of 50 s 45 kpsi and 10 s 14.7 psi; cell line samples: 120 cycles of 20 s 45 kpsi and 10 s 14.7 psi) at 35 °C. Whole cell/ tissue lysates were then sonicated for 25 s with 1 min interval on ice for 4 times. After removing tissue debris or unbroken cells, if any, by centrifugation, protein lysates were reduced and alkylated prior to proteolytic digestion. Lys-C (enzyme to substrate ratio: 1:50) and trypsin (1:30) were sequentially added to digest the proteins. Digestion was accelerated under a PCT scheme of 50 s 25 kpsi and 10 s 14.7 psi (cell line samples: 25 s 25 kpsi, 10 s 14.7 psi for 45 mins), under which both Lys-C and trypsin remain active. Lys-C digestion was performed in 6 M urea for 45 cycles, whereas trypsin digestion was performed in further diluted urea (1.6 M) for 90 cycles (cell line samples: 24 s 25 kpsi, 10 s 14.7 psi for 90 min). Subsequently, trifluoroacetic acid (TFA) was added to a final pH of around 2 before C18 desalting using SEP-PAK C18 cartridges (Waters Corp., Milford, MA, USA).

#### Off-gel electrophoresis (OGE)

After digestion and desalting steps, clean peptides were re-solubilised in OGE buffer, which contained 5% (v/v) glycerol, 0.7% ACN and 1% (v/v) carrier ampholytes mixture (IPG buffer pH 3.0–10.0, GE Healthcare). The peptides were separated on a 3100 OFFGEL (OGE) Fractionator (Agilent Technologies) using a 24 cm pH 3–10 IPG strip (GE Healthcare) at a maximum of 8,000 V, 50 μA, and 200 mW until 50 kVhrs were reached. After all fractions were recovered, they were desalted on C18 reversed-phase MicroSpin columns (The Nest Group Inc.) and pooled according to the following schemes for MS injections:

HEK293

pool 1 (fraction 1–2), pool 2 (fraction 3), pool 3 (fraction 4), pool 4 (fraction 5), pool 5 (fraction 6–7), pool 6 (fraction 8–9), pool 7 (fraction 10–11), pool 8 (fraction 12–16), pool 9 (fraction 17–18), pool 10 (fraction 19–21), pool 11 (fraction 22–24).

NCI60 panel

pool 1 (fraction 1–2), pool 2 (fraction 3), pool 3 (fraction 4), pool 4 (fraction 5), pool 5 (fraction 6–7), pool 6 (fraction 8–9), pool 7 (fraction 10–11), pool 8 (fraction 12–15), pool 9 (fraction 16–19), pool 10 (fraction 20–21), pool 11 (fraction 22), pool 12 (fraction 23–24).

THP-1

No pooling was done. Each of the 24 fractions was injected once except for fraction 3, 4, 9, and 22, which were injected twice.

#### 1D gel electrophoresis (1D GE)

A pool of 18 kidney tissue samples was resolved into 15 gel fractions based on the molecular mass of proteins using SDS-PAGE^34^. These fractions were digested independently in-gel before mass spectrometric analysis using standard protocol^35^.

#### Strong anion exchange (SAX)

A total of 50 μg of peptides was separated on a pipet-based anion exchanger, which was assembled following the StageTip principle by stacking 6 layers of a 3 M Empore Anion Exchange disk (Varian, 1214−5012) into a 200 μl micropipet tip, as previously described^36^. Briefly, the equilibration buffer was composed of 20 mM acetic acid, 20 mM phosphoric acid and 20 mM boric acid was titrated with NaOH to the desired pH. Peptides were loaded at pH 11 and fractions were subsequently eluted with buffer solutions of pH 8, 6, 5, 4, and 3, respectively by centrifugation at 7,000 × *g* each time. The flow-through and the five pH-eluted fractions were all captured on C18 StageTips.

#### RT normalization peptides

For the RT normalization and analysis, the peptides from the iRT Kit (Biognosys AG, Schlieren, Switzerland) were added to all samples prior to MS injection according to vendor instructions^37^.

### DDA mass spectrometry for spectral library generation

For spectral library generation, an AB SCIEX TripleTOF 5600+ System mass spectrometer was used. It was operated essentially as described before^23,24^: All samples were analyzed on an Eksigent nanoLC (AS-2/1Dplus or AS-2/2Dplus) system coupled with a SWATH-MS-enabled AB SCIEX TripleTOF 5600+ System. The HPLC solvent system consisted of buffer A (2% acetonitrile and 0.1% formic acid in water) and buffer B (2% water with 0.1% formic acid in acetonitrile). The samples were separated in a 75 μm-diameter PicoTip emitter (New Objective) packed with 20 cm of Magic 3 μm, 200 Å C18 AQ material (Bischoff Chromatography). The loaded material was eluted from the column at a flow rate of 300 nl/min with the following gradient: linear 2–35% B over 120 min, linear 35–90% B for 1 min, isocratic 90% B for 4 min, linear 90–2% B for 1 min and isocratic 2% solvent B for 9 min. The mass spectrometer was operated in DDA top20 mode, with 500 and 150 ms acquisition time for the MS1 and MS2 scans respectively, and 20 s dynamic exclusion. Rolling collision energy with a collision energy spread of 15 eV was used for fragmentation.

### Spectral and assay library generation

All raw instrument data (Data Citation 1) were centroided and processed as described previously^24,27^. The assay library was generated according to the following protocol: The TPP^38^ (4.6.0) and SpectraST^39^ (5.0) were used for the analysis of the shotgun proteomics runs. The datasets were searched individually using X!Tandem^40^ (2011.12.01.1) with k-score plugin^41^, Myrimatch^42^ (2.1.138), OMSSA^43^ (2.1.8) and Comet^44^ (2013.02r2) against the full non-redundant, canonical human genome as annotated by UniProtKB/Swiss-Prot^45^ (2014_02) with 20 270 ORFs and appended iRT peptide and decoy sequences. Carbamidomethyl (C) was used as a fixed modification; oxidation (M) was the only variable modification. Parent mass error was set to ±50 p.p.m., fragment mass error was set to ±0.1 Da. The search identifications were then combined and statistically scored using PeptideProphet^46^ and iProphet^47^ within the TPP^38^. MAYU^48^ (1.07) was used to select an iProphet cutoff of 0.999354, resulting in a protein FDR of 1.03%. SpectraST was used in library generation mode with CID-QTOF settings and iRT normalization at import against the iRT Kit peptide sequences (-c_IRTirtkit.txt -c_IRR) and a consensus library was consecutively generated^49^. The script spectrast2tsv.py (msproteomicstools 0.2.2; https://pypi.python.org/pypi/msproteomicstools) was then used to generate the asay library with suggested settings: -l 350,2000 -s b,y -x 1,2 -o 6 -n 6 -p 0.05 -d -e -w swath32.txt -k openswath. The OpenSWATH (OpenMS/develop, revision: 03377b6) tool ConvertTSVToTraML converted the TSV file to TraML and decoys were appended to the TraML assay library with the OpenSWATH tool OpenSwathDecoyGenerator as described before^24^ in reverse mode with a similarity threshold of 0.05 Da and an identity threshold of 1. The assay library (Data Citation 2) was further converted to table format compatible with OpenSWATH, PeakView, Skyline and Spectronaut.

### DIA mass spectrometry (SWATH-MS)

For SWATH-MS data acquisition (Data Citation 3), the same mass spectrometer and LC-MS/MS setup was operated essentially as described before^23,24^, using 32 windows of 25 Da effective isolation width (with an additional 1 Da overlap on the left side of the window) and with a dwell time of 100 ms to cover the mass range of 400–1,200 *m/z* in 3.3 s. Before each cycle, an MS1 scan was acquired, and then the MS2 scan cycle started (400–425 *m/z* precursor isolation window for the first scan, 424–450 *m/z* for the second... 1,174–1,200 *m/z* for the last scan). The collision energy for each window was set using the collision energy of a 2+ ion centered in the middle of the window with a spread of 15 eV.

### SWATH-MS data analysis

#### OpenSWATH

An improved development version of the OpenSWATH (OpenMS/develop, revision: 03377b6) analysis workflow (OpenSwathWorkflow) (http://www.openswath.org) was used for all data analyses. The parameters were selected analogously to the ones described before^24^: min_rsq: 0.95, min_coverage: 0.6, min_upper_edge_dist: 1, mz_extraction_window: 0.05, rt_extraction_window: 600, extra_rt_extraction_window: 100.

pyprophet (0.9.2) (https://pypi.python.org/pypi/pyprophet) was run on the OpenSwathWorkflow output adjusted to contain the previously described scores (xx_swath_prelim_score, bseries_score, elution_model_fit_score, intensity_score, isotope_correlation_score, isotope_overlap_score, library_corr, library_rmsd, log_sn_score, massdev_score, massdev_score_weighted, norm_rt_score, xcorr_coelution, xcorr_coelution_weighted, xcorr_shape, xcorr_shape_weighted. yseries_score)^24^ and proteotypic peptides only with enabled MAYU export and 30-fold semi-supervised learning iterations. This generated an OpenSWATH peptide identification list, a FASTA library containing only the targeted peptides and proteins and the false target:decoy ratio (the ratio of targets which could not be detected and decoys) for direct analysis with MAYU.

MAYU (1.07) was used with a maximum mFDR of 0.1, 200 mFDR steps and the calculated false target:decoy ratio to compute assay-level q-value (m_score) cutoffs corresponding to the selected protein FDR. All further analyses were conducted on per run individually analyzed and filtered peptide and protein identifications.

#### PeakView

A previously collected data set of AP-SWATH samples was reprocessed using PeakView (AB SCIEX) as described by Lambert *et al.*^28^ Essentially the raw data was processed using the sample-specific assay library or the combined assay library, extracting peak areas and scoring using the PeakView SWATH micro app. Peak areas were extracted and filtered to remove all peptides, which do not have a single measurement with an FDR less than 1% across all measurements.

The extracted peak areas were processed through most likely ratio normalization and fold change determination as described before^28^. The results for the fold change analysis from the sample-specific assay library were compared to the fold-change results from the combined assay library.

## Data Records

### Data Record 1

The mass spectrometry discovery proteomics data (instrument raw files, centroided mzXML and identified peptides in pepXML report) used to generate the combined assay library have been deposited to the ProteomeXchange Consortium (http://proteomecentral.proteomexchange.org) via the PRIDE partner repository^51^ with the dataset identifier PXD000953 (Data Citation 1).

### Data Record 2

The spectral libraries (SpectraST format) and assay libraries (CSV, TraML) are available for different SWATH-MS data analysis tools at the SWATHAtlas with the dataset identifiers SAL00016-35 (Data Citation 2).

### Data Record 3

The mass spectrometry SWATH-MS data (instrument raw files, mzXML and identified peptides in OpenSWATH report) used to validate the sample-specific and combined assay libraries have been deposited to the ProteomeXchange Consortium (http://proteomecentral.proteomexchange.org) via the PRIDE partner repository^50^ with the dataset identifier PXD000954 (Data Citation 3).

## Technical Validation

### Assay library saturation analysis

**Figure 2:**
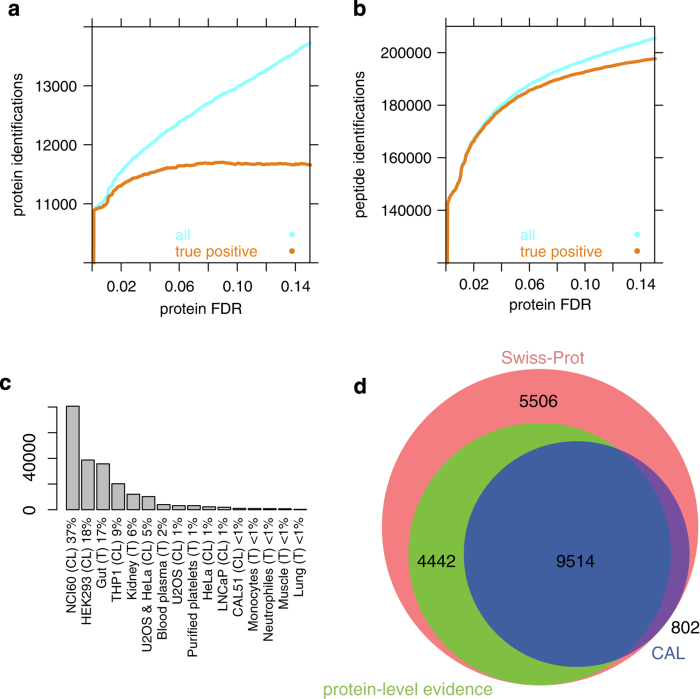
Statistics of the combined assay library and comparison to other human proteome mapping efforts. (**a**) True positive (red) and all protein identifications (blue) as a function of protein FDR. The graph indicates that the number of true positive protein identifications saturates at a protein FDR cutoff of 0.05. Additional identifications at less strict FDR cutoffs are mainly false positive protein identifications. (**b**) True positive (red) and all peptides identifications (blue) as a function of protein FDR. The graph indicates that the number of true positive peptide identifications correlates strongly with the total number of peptide identifications and does not reach saturation within typical levels of protein FDR cutoffs. (**c**) The number of PSM per sample type contributed to the assay library. Multiple PSM can constitute a consensus spectrum and are individually counted per MS injection. The NCI60 cell line panel contributed most, and HEK293 cells, gut tissue and THP1 cells each contributed to more than 10% of all spectra. (**d**) Overlap of human proteins curated by UniProtKB/Swiss-Prot, a subset annotated with protein-level evidence and the presented combined assay library (CAL). On the protein level, the assay library provides 68.2% coverage of the proteins with evidence while providing assays for an additional 802 proteins. Compared to UniProtKB/Swiss-Prot, the assay library contains 50.9% of all 20,264 proteins.

Large-scale MS-based proteomics experiments are prone to accumulation of false identifications, both at the peptide and protein level. It is thus crucial to filter these datasets restrictively, especially for the purpose of assay library generation. We applied the strategy implemented in MAYU^48^ to adjust the assay library to an FDR of 1% at the protein level, resulting in an iProphet^47^ probability cutoff of 0.999354. At this cutoff, the number of true positive protein identifications already reaches saturation (Figure 2a). This is in contrast to the number of true positive peptide identifications, which could be further increased at the cost of accepting a higher number of false positive protein identifications (Figure 2b). This result is in line with observations from other large-scale datasets, where the true detectable proteins generally have many associated peptides that match redundantly to the same protein. The false positive identifications on the other hand do not show this redundancy and thus the error-rate needs to be controlled very strictly, resulting in a number of false negative identifications^51^.

The number of proteins identified from a DDA dataset depends significantly on the redundancy of the sequence database searched. Databases with a high degree of sequence redundancy can inflate the protein identifications because substantially similar or indistinguishable proteins are counted as separate species. Therefore, the application of redundant protein databases like UniprotKB/TrEMBL or the International Protein Index (IPI) is not recommended for the purpose of assay library generation because of their increased potential for generating random single hit identifications^48,52^. For this study, we used UniprotKB/Swiss-Prot as basis for protein annotation, which is considered to be the leading universal curated protein sequence database^45,53^ and which contains only non-redundant entries.

The combined assay library (CAL) contains injections from 16 different sample types and the relative contribution of each sample to the consensus spectral library varies from below 1 to 37%. In general, the NCI60 cell line panel, the HEK293 and THP1 cell lines and gut and kidney tissue samples were the major contributors, collectively accounting for close to 90% of all consensus peptide spectrum matches (PSM) above the threshold (Figure 2c). This large coverage is mainly due to extensive fractionation on the protein and peptide level and the large number of MS injections per sample type.

### Relation to present state of proteome discovery

In recent years, several studies and projects have aimed at mapping the complete human proteome, among them the HUPO Chromosome-centric Human Proteome Project (C-HPP)^7,8^, which attempts to characterize at least one protein product for each human protein-coding gene^54^. The proteomes of several human cell lines have been exhaustively identified^4–6,4–6,4–6^ and recently, draft maps of the human proteome have been published, accounting for 84%^9^ or 92%^10^ of the annotated human genome.

We compared the proteins contained in the combined assay library with the proteins annotated by UniProtKB/Swiss-Prot (version 2014_05) and the proteins annotated in there with evidence on protein-level^55^. We mapped the non-redundant, canonical list of UniProtKB/Swiss-Prot identifiers to the proteins identified by proteotypic peptides contained in the combined assay library. Figure 2d indicates that on the protein level, our library reaches 68.2% coverage of the 13,956 proteins annotated with protein-level evidence, while providing assays for an additional 802 proteins. Compared to UniProtKB/Swiss-Prot, the combined assay library contains 50.9% of all 20,264 proteins. Table 2 provides an overview of the contents of the combined assay library.

### Applicability of the combined assay library for SWATH-MS targeted data analysis

**Figure 3:**
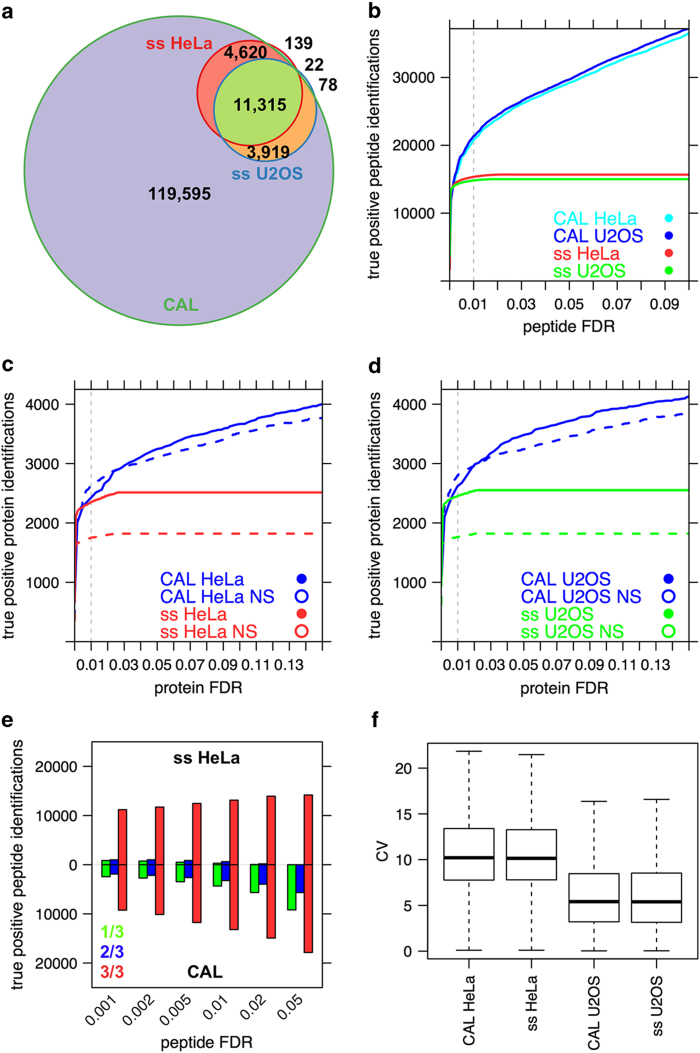
Number of peptide and protein identifications by SWATH-MS using different proteotypic assay libraries. (**a**) The proteotypic peptides contained in the combined assay library (CAL) and the sample–specific (ss) assay libraries and their overlap is depicted. The overlap on peptide-level between the sample-specific libraries is more than 70% and around 80% on protein-level. 239 peptides contained in the sample-specific libraries were not included in the CAL, since they did not meet the stricter quality cutoff of the CAL. (**b**) The number of true positive peptide identifications in dependency of the peptide FDR is depicted. Using the combined library, the number of true positive peptide identifications matches the sample-specific libraries at peptide FDR below 1% (dashed grey line). (**c**,**d**) The number of true positive protein identifications of a HeLa (**c**) or U2OS (**d**) whole cell lysate in a single, unfractionated injection in dependency of the protein FDR is depicted. Protein FDR cutoffs are either reported for all identifications or non-single hits (NS). The CAL provides similar sensitivity compared to the sample-specific libraries for HeLa and U2OS at typical levels of error-rate control. The non-single hit identifications of the CAL generally provide a higher sensitivity at lower protein FDR cutoffs. The dashed grey line indicates the protein FDR cutoff at 1%. (**e**) Reproducibility of the peptide identifications in dependency of the peptide FDR. The colors indicate reproducibility in 1 (green), 2 (blue) or 3 (red) of 3 technical replicates. Both ss HeLa (top) and CAL (bottom) enable detection of a similar number of assays among all replicates at the same peptide FDR. The CAL enables detection of more low intensity peptides in only one or two replicates. (**f**) Distribution of the coefficient of variation (CV) of summed transition intensities of precursors identified in all three replicates at 1% peptide FDR. The median CV of 5% (U2OS) to 10% (HeLa) corresponds well with the expected technical variation and is very similar between sample-specific and the combined assay library.

An analysis using whole cell digest samples from HeLa and U2OS cell lines was conducted to compare the performance of the combined (CAL) and sample-specific assay libraries (ss HeLa/ss U2OS). First, we generated sample-specific assay libraries from lysates of the respective cell lines by acquiring DDA datasets (which are also contained in the combined assay library) from three repeat injections of the unseparated peptide samples. For the HeLa cells the resulting sample-specific assay library contained 2,583 proteins, 16,096 peptides, 18,124 precursor ion sequences and 108,744 transitions. For the U2OS cells the library contained 2,610 proteins, 15,334 peptides, 17,360 precursors and 104,160 transitions. For both cell lines the data were filtered to a protein FDR of 1% and only proteotypic assays were considered for all further analyses. The overlap with the combined assay library was found to be over 99% on both peptide and protein level for both cell lines (Figure 3a). The overlap between the two sample-specific libraries is on peptide-level more than 70% and about 80% on protein-level. Both libraries were used to individually analyze the same sample acquired in DIA mode using OpenSWATH^24^. The q-value threshold (m_score) on assay level was used to estimate the protein FDR as described above.

At a protein FDR of 1%, the number of true positive protein identifications from a sample is very similar when the whole combined assay library or sample-specific assay libraries were used (Figure 3c,d). However, compared to the number of the non-single hits identified by the sample-specific assay libraries, the combined assay library provides an increased protein-level coverage of 49–59% (Table 3). This apparent discrepancy can be resolved in context of the number of peptides that are identified as true positives using the combined assay library compared to the sample-specific assay libraries. Because the combined assay library enables detection of over 35% more peptides at a peptide FDR of 1% (Figure 3b), excluding single hits enables detection of more proteins. Overall, these data show that the combined assay library identifies peptides at a higher level of sensitivity at typical levels of FDR control.

The reproducibility of the peptide identifications among three technical replicates in dependency of the peptide FDR for the HeLa samples is depicted in Figure 3e. The number of peptides identified in all three samples is similar for both the combined and sample-specific libraries. However, the CAL detected a higher number of peptides in only one or two replicates. Further assessment of these peptides at 1% FDR for the CAL and sample-specific library indicates that they are mainly low-intensity peptides (CAL: 1/3 (detected in 1 out of 3 replicates) 33,433±38,083 (mean±s.d. of summed fragment ion intensities per precursor), 2/3 (39,504±39,440), 3/3 (89,935±140,914); ss HeLa: 1/3 (35,865±38,467), 2/3 (39,440±52,346), 3/3 (97,226±152,470)). The majority (CAL: 77.4%; ss HeLa: 82.0%) of proteins mapped by these low-intensity peptides were also detected by different, higher-intensity peptides in all three replicates. This indicates that the assays are not resulting in false positive protein identifications, but rather enable measuring of additional peptides of the same proteins and that the assays of the CAL and sample-specific assay libraries are very similar in terms of reproducibility of identification in targeted proteomics experiments. These assays are not present in the sample-specific assay libraries due the sample complexity and limitations of the DDA algorithms that only select the most intense precursors for fragmentation.

The coefficient of variation (CV) of the quantified signals on precursor level was found to correspond well with the expected technical variation between replicates of below 20%^24^ (Figure 3f). Further, the CV of the quantified signals using the combined and sample-specific libraries are very similar for the two cell lines, indicating conserved reliable quantification performance.

### Portability of the combined assay library to different sample types and laboratories

**Figure 4:**
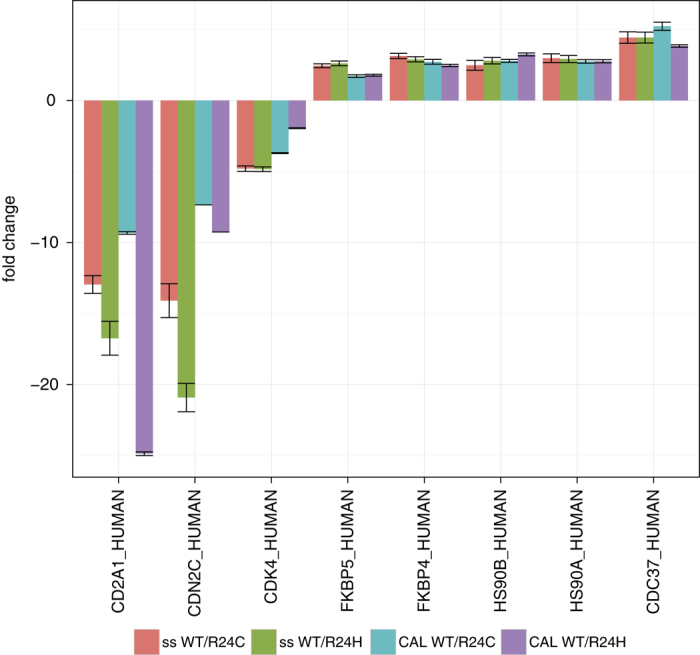
Application of the combined assay library (CAL) to an independently acquired dataset (CDK4 AP-SWATH, Lambert *et al.*^28^) and comparison to the sample-specific assay library (ss). The fold changes of the comparison wild type (WT) and mutants (R24C or R24H) with whiskers for standard deviation are indicated. The assays contained in the combined library for CD2A1 and CDN2C covered fewer and different peptides than the sample-specific assay library and thus the fold change is smaller. The results indicate that comparable qualitative and quantitative results using the combined assay library can be retrieved from SWATH-MS experiments conducted using different experimental setups, data acquisition and data analysis strategies.

To test the portability of the generated assay library we used a subset of assays for specific proteins from the combined assay library for reanalysis of the CDK4 AP-SWATH dataset of Lambert *et al.*^28^. This dataset was generated on the same type of instrument used for the generation of the assay library presented here. However, the SWATH-MS data and the DDA data used to generate a sample-specific library were acquired in a different laboratory, at a different time point and using different chromatographic conditions. Using either the original sample-specific library or the corresponding assays contained in the combined library reported here we determined the fold change of the proteins between the wild type and the mutant CDK4 states (R23C, R23H). Figure 4 shows the comparison and overlap of the original analysis and reanalysis using the assays from the combined library. The protein fold change measurements between the different assay libraries are comparable. The data therefore indicate that the assays contained in the combined library can be used successfully to perform protein quantification even if the data were acquired at different times and in different laboratories. Investigation of the peptides within the combined in comparison to those in the sample-specific assay library created as part of the original publication showed that in most cases there was equivalent coverage of proteins between different libraries. In those cases where protein expression profiles were different between the different assay libraries as in CD2A1 and CDN2C, the difference in the fold change can be attributed to the difference in the number of peptides present within the library. These results demonstrate that the assays contained in the combined assay library presented here are portable between different experimental setups.

## Usage Notes

### Application of the assay library to SWATH-MS data

There are two different ways to apply the assay library to search SWATH-MS datasets. The first is a selective search for predetermined sets of proteins and the second is a comprehensive search of a SWATH-MS map with the whole library. In the first case a selection of peptides or proteins of interest is available as *prior* information, e.g., from earlier proteomics or transcriptomics measurements or from the literature. The combined assay library can thus be filtered accordingly so that the query transition list only contains assays for these targeted proteins or peptides. To simplify this step, we provide querying of the combined assay library for specific proteins and peptides on the SWATHAtlas. These assays can be used in software like Skyline^25^ or PeakView for data analysis and visualization.

In the second case there is no pre-selection of target peptides or proteins and the whole assay library is used to search a SWATH-MS map by an automated software like OpenSWATH^24^. Since the whole combined library contains assays for more than 10,000 proteins and a typical short gradient single SWATH-MS map will typically lead to the identification of 2,000–5,000 proteins, most proteins targeted by the whole assay library will either not be present in the sample or not be detectable. To avoid false positives due to the multiple comparisons problem, it is critical to appropriately set score cutoffs according to the peptide or protein FDR with tools like MAYU^48^. This approach is dependent on the proper application of the target-decoy approach^56^ and we have found that especially for very large assay libraries as the one presented here, it is crucial to generate decoy assays that both are guaranteed to be different from the target assays and that represent the full sample. To enable generation of decoy transitions for even highly repetitive or palindromic peptide sequence, we found that full reversion of the sequences fulfills these requirements.

The effect of the multiple comparison problem is illustrated by the application of the whole combined assay library to the HeLa SWATH-MS datasets described above. In the analyses MAYU determined an assay FDR of approximately 0.0036% resulting in a protein FDR of 1%. In comparison, for a sample-specific library, the same protein FDR was reached with an assay FDR of about 0.6%. This discrepancy is partially related to the observation in shotgun proteomics database searching that searching very large databases, e.g., six-frame translations of genomic databases, increases the chances of random PSMs. However, the situation differs from sequence database searching in that the targeted approach attempts to detect specific signal groups in a variable number of experimentally observed ion chromatograms.

An updated version of OpenSWATH is provided (http://www.openswath.org) that directly enables protein FDR assessment using MAYU.

The presented data was acquired on Eksigent nanoLC (AS-2/1Dplus or AS-2/2Dplus) systems coupled with an AB SCIEX TripleTOF 5600+ system and the combined assay library is therefore optimized for this type of instrument. However, the assay library could also be applied to DIA data acquired on other high-resolution instruments. In such a case, the expected fraction of detectable assays is depending on the similarity of the instrumentation in terms of fragmentation method and liquid chromatography. Particularly, when qTOF-CID spectra, as the ones presented here, are compared to ion trap HCD spectra, the conservation of the fragment pattern is high, indicating good portability of the assays^57,58^. Further, the normalized retention time used here is a dimensionless value that can be transformed to different LC setups using spiked-in standards^37^. Finally, the semi-supervised learning approach employed by mProphet^59^ and related software like OpenSWATH, Spectronaut and Skyline adapts the influence of potentially decreased fragmentation or retention time conservation on the discriminant scoring function to maintain accurate separation of true and false detected assays.

### Generation of custom assay libraries from the presented data

Custom assay libraries can be optimized for specific sample types, proteoforms and proteomic background. For special applications such as the analysis of proteoforms, custom assay libraries can be generated by searching the spectral data additionally for post-translational modifications such as phosphorylation or by using a different protein sequence database, e.g., one containing protein isoforms. It is recommended to apply an assay library generation workflow that is scalable and enables control of the error rate. A manuscript providing detailed instructions for the generation of large-scale assay libraries is in preparation by the authors (Schubert, O. T., Gillet, L. C., Collins, B. C., Navarro, P., Rosenberger, G., Wolski, W. E., Lam, H., Amodei, D., MacLean, B., Mallick, P. & Aebersold, R.). Particularly for modifications, the confidence for correct site assignment needs to be assessed and accounted for ref. 60.

The transitions of the combined assay library have been selected according to a protocol that enables qualitative and quantitative comparable results as sample-specific assay libraries (Figures 3 and 4). Assays with many interfered transitions can be detected automatically by the software tools used in this study and rather affect the sensitivity than the selectivity and thus do not increase the number of false positives^24^. Because the combined assay library contains assays for more than one proteotypic peptide for 86.5% of all proteins, a different peptide can be used for quantification in most such cases. However, for certain applications, especially when analysis of very complex human samples or differentially site-modified proteoforms is conducted, the transition selection could be altered according to the unique ion signature (UIS) concept^61^. Using tools like SRMCollider^62^, transitions could be selected for a given background proteome (e.g., based on previously identified proteins) to minimize potential interferences with other co-eluting peptides. Additionally, SWATH-MS enables iterative reanalysis using different assays for the same peptide and thus the combined assay library could be optimized for a particular sample type using empirical criteria.

### Extension of the human assay library

This is a first edition of the combined human SWATH-MS assay library and further extensions will be added. Analogous to the HUPO Human Proteome Project and the recent studies mapping the human proteome^9,10^, data fulfilling the requirements for SWATH-MS assay library generation can be collected in public repositories like ProteomeXchange^63^ and periodically, new builds of the assay library can be generated as new datasets covering extended parts of the human proteome become available. As demonstrated in this study, the extension will not compromise results derived from subsets of the assay library but enable a more complete and comparable targeted analysis of human SWATH-MS datasets.

## Additional information

**How to cite this article:** Rosenberger, G. *et al.* A repository of assays to quantify 10,000 human proteins by SWATH-MS. *Sci. Data* 1:140031 doi: 10.1038/sdata.2014.31 (2014).

## Supplementary Material

Supplementary Information

## Figures and Tables

**Table 1:** Overview of the contents of the combined assay library.

**Sample type**	**Protein fractionation**	**Proteolytic digestion**	**Peptide fractionation**	**MS injections**
HEK293 (CL)	AP (Kinases)	Trypsin	None	12
HEK293 (CL)	AP (14-3-3)	Trypsin	None	29
HEK293 (CL)	SEC	Trypsin	None	81
HEK293 (CL)	None	Trypsin	OGE	11
HEK293 (CL)	None	Trypsin	None	1
U2OS (CL)	None	PCT	None	13
HeLa (CL)	None	PCT	None	9
U2OS and HeLa (CL)	None	Trypsin	OGE	24
NCI60 (CL)	None	PCT	None	13
NCI60 (CL)	None	Trypsin	OGE	24
CAL51 (CL)	None	Trypsin	None	5
CAL51 (CL)	None	Trypsin	1D GE	2
THP1 (CL)	None	Trypsin	OGE	27
LNCaP (CL)	None	Trypsin	SAX	6
LNCaP (CL)	None	Trypsin	None	1
Kidney (T)	None	Trypsin	1D GE	15
Kidney (T)	None	PCT	None	16
Large intestine (T)	None	Trypsin	OGE	24
Muscle (T)	None	PCT	None	3
Lung (T)	None	PCT	None	2
Blood plasma (T)	None	Trypsin	SAX	8
Monocytes (T)	None	Trypsin	None	1
Neutrophils (T)	None	Trypsin	None	1
Purified platelets (T)	None	Trypsin	None	3
Total	**331**			
CL refers to cell line and T refers to tissue, indicating the source of the specimen. The full sample annotation is provided in Supplementary Table 1.				

**Table 2:** Assay statistics of the combined assay library.

	**Proteotypic**	**Proteotypic+Shared**
Proteins	10,316	11,588
Peptides	139,449	146,576
Precursors	194,052	204,545
Transitions	1,164,312	1,227,270
The number of proteins, peptides, precursors and transitions, filtered at protein FDR 1% is depicted. The combined assay library is provided with all target and decoy assays, but only proteotypic assays were considered for all downstream analysis.		

**Table 3:** Identification statistics of the combined and sample-specific assay libraries.

**Protein FDR**	**CAL HeLa**		**ss HeLa**		**CAL U2OS**		**ss U2OS**	
	**prot**	**pep**	**prot**	**pep**	**prot**	**pep**	**prot**	**pep**
1%	2,417	14,930	2,353	14,635	2,617	15,608	2,452	14,360
2%	2,730	17,294	2,467	15,416	2,989	18,321	2,541	14,982
5%	3,246	21,128	2,514	15,672	3,486	21,893	2,552	15,003
NS 1%	2,608	23,075	1,750	14,999	2,803	24,009	1,763	14,599
NS 2%	2,804	25,005	1,798	15,537	2,965	25,497	1,815	15,002
NS 5%	3,111	28,002	1,820	15,668	3,241	28,442	1,819	14,999
The number of identified proteotypic peptides and proteins in SWATH-MS datasets of whole cell lysates of HeLa and U2OS cell lines at commonly used protein FDR cutoffs using combined (CAL) and sample-specific (ss) assay libraries is reported. Protein FDR cutoffs are either reported for all identifications or non-single hits (NS). The true positive protein (prot) and peptide (pep) identifications for the combined assay library and sample-specific assay libraries are reported as estimated by MAYU.								
